# Supplementary material for: DMRforPairs: identifying Differentially Methylated Regions between unique samples using array based methylation profiles
Source: BMC Bioinformatics. 2014 May 15;15:141. doi: 10.1186/1471-2105-15-141 (PMC4046028; doi:10.1186/1471-2105-15-141)
Supplement: Additional file 2 — DMRforPairs output for the comparison of A431-MCF7 and NA17018-NA17105. Please start from the HTML files in each folder. Available via the BMC Bioinformatics website. [file 1471-2105-15-141-S2.zip › 1394847754114233_MOESM2_ESM/A431_MCF7/figures/26516.pdf]

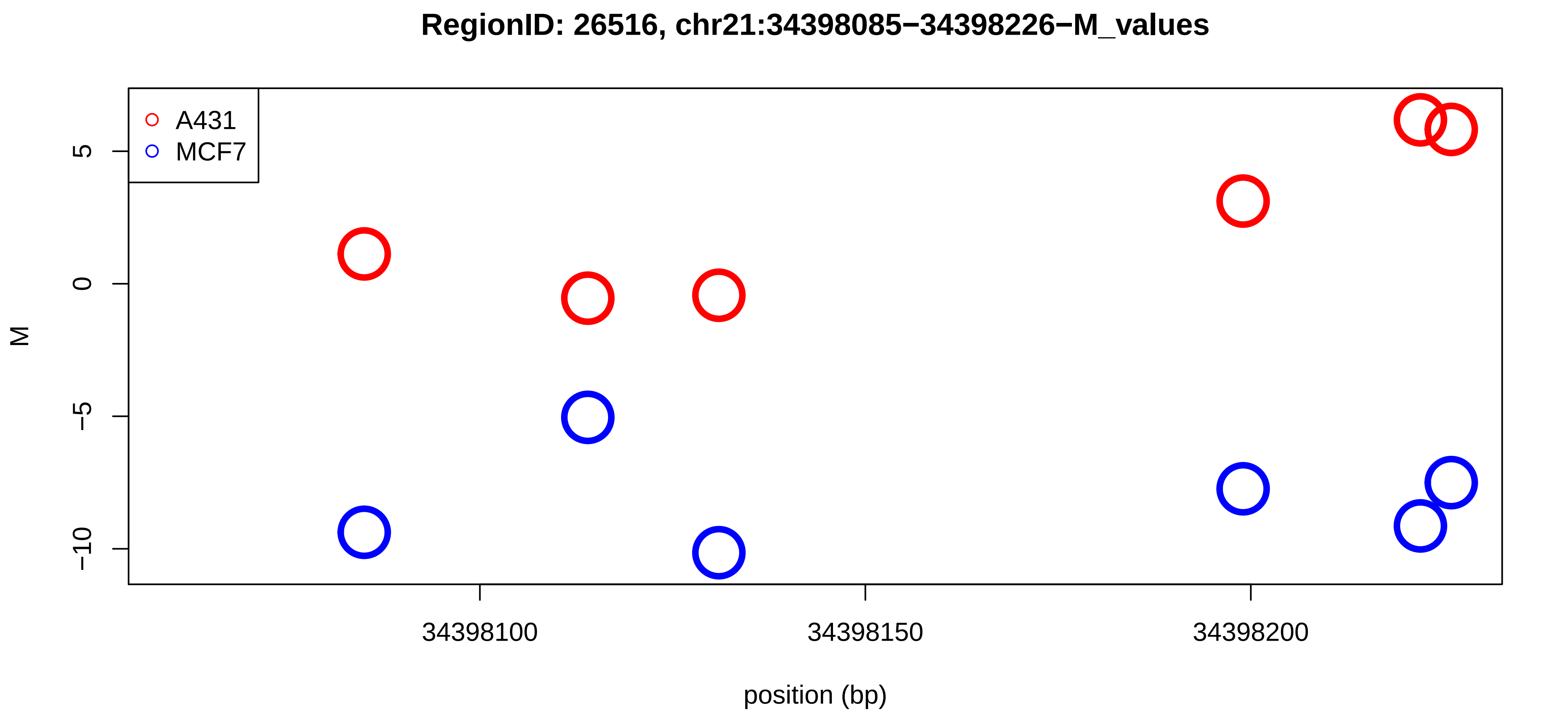
RegionID: 26516, chr21:34398085−34398226−M_values
A431
MCF7
5
0
−5
−10
M
34398100
34398150
34398200
position (bp)

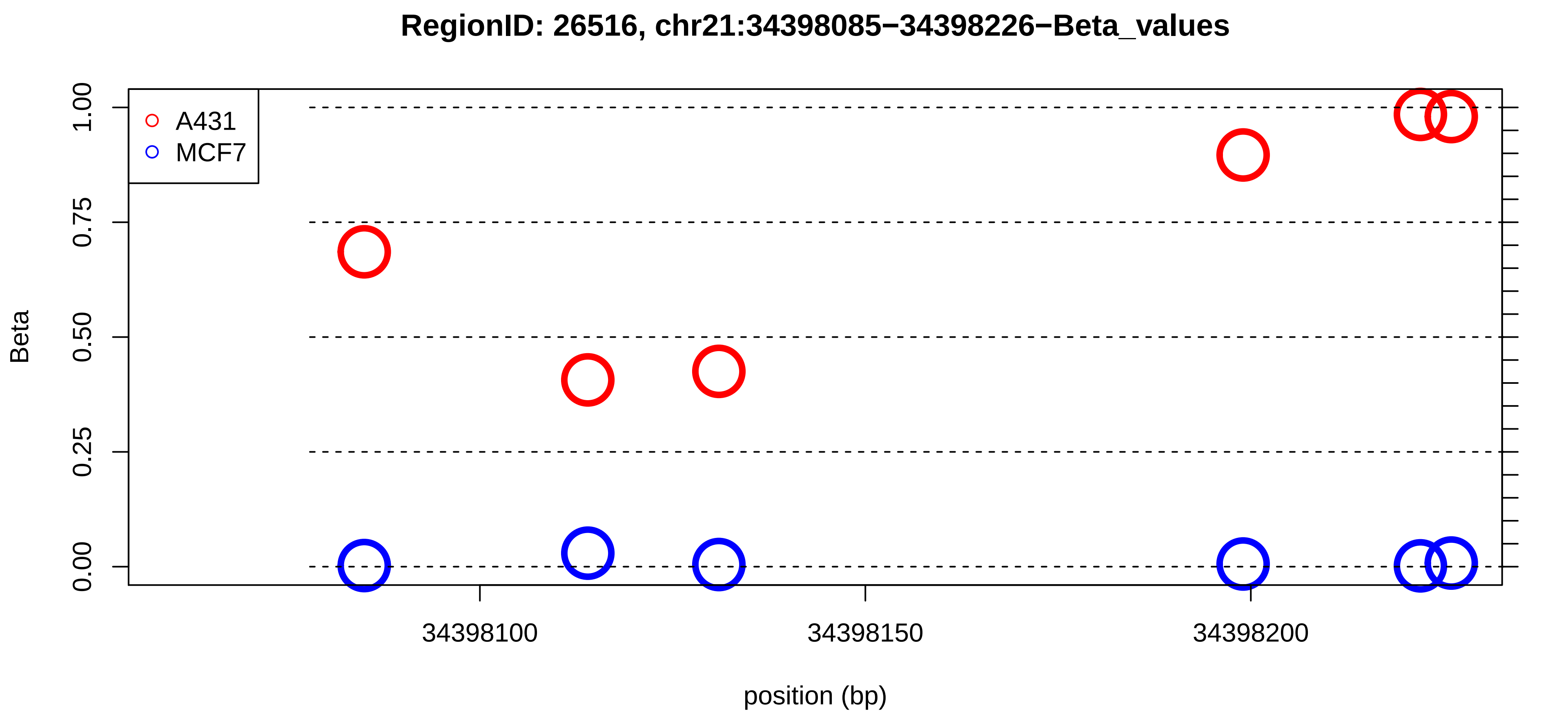
RegionID: 26516, chr21:34398085−34398226−Beta_values
A431
MCF7
1.00
0.75
0.50
0.25
0.00
Beta
34398100
34398150
34398200
position (bp)

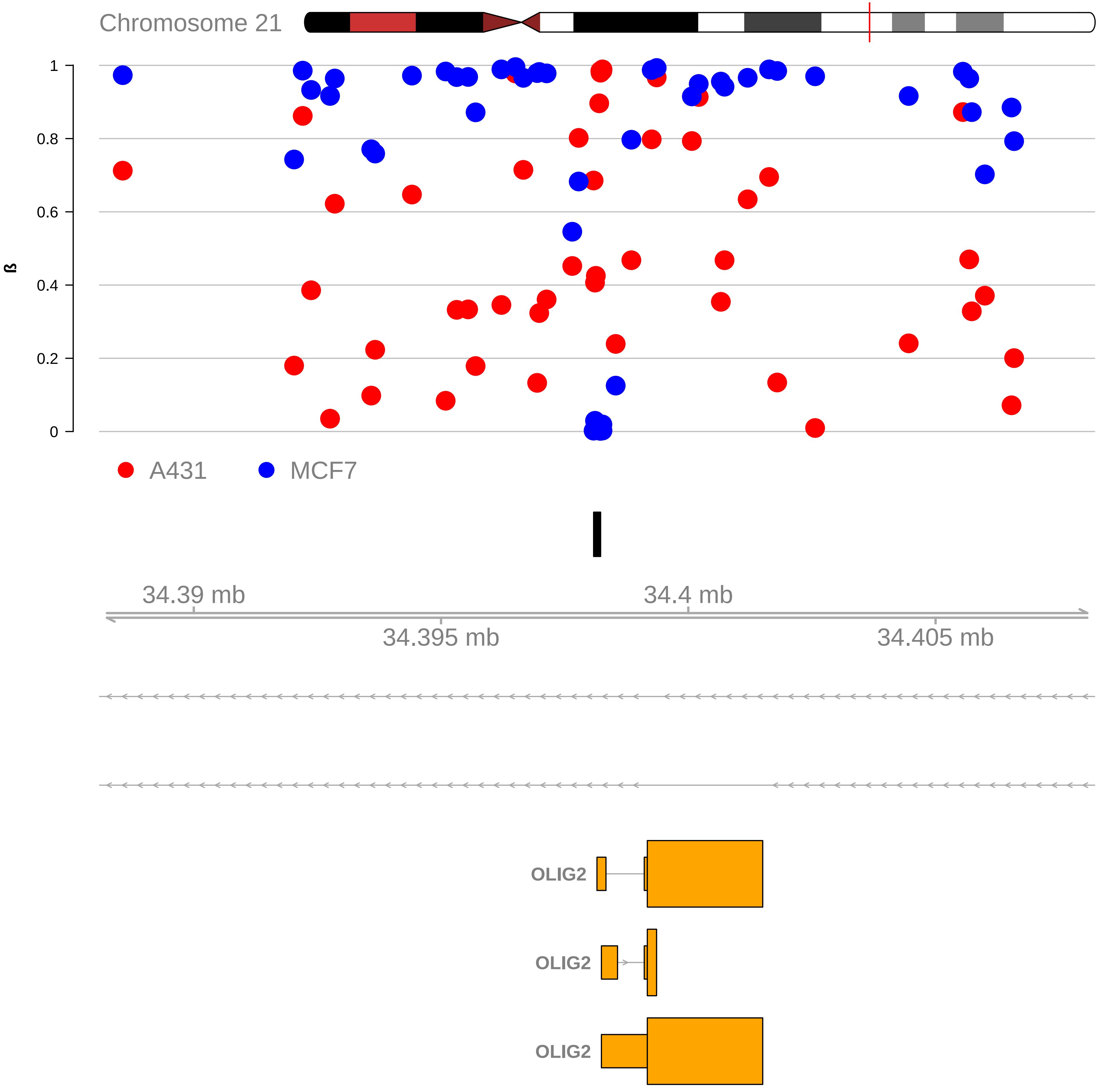

Chromosome 21
ß
1
0.8
0.6
0.4
0.2
0
A431
MCF7
34.39 mb
34.4 mb
34.395 mb
34.405 mb
OLIG2
OLIG2
OLIG2
